# Shelf Life Extension of Fresh Buffalo Meat Using Spice Powders and Lavender Essential Oil During Storage Under Refrigeration

**DOI:** 10.3390/foods15050947

**Published:** 2026-03-07

**Authors:** Athanasia P. Marangeli, Vassilios K. Karabagias, Glykeria E. Angelaki, Dimitrios G. Lazaridis, Nikolaos D. Andritsos, Olga Malisova, Ioannis K. Karabagias

**Affiliations:** Department of Food Science & Technology, School of Agricultural Sciences, University of Patras, G. Seferi 2, 30100 Agrinio, Greece; athanasiamaragelis@gmail.com (A.P.M.); vkarampagias@upatras.gr (V.K.K.); glykeriaangelaki@gmail.com (G.E.A.); dlazaridis@ac.upatras.gr (D.G.L.); nandritsos@upatras.gr (N.D.A.); omalisova@upatras.gr (O.M.)

**Keywords:** buffalo meat, PA/PE packaging, refrigeration, spices, essential oils, antibacterial activity, shelf life extension

## Abstract

We studied the shelf life of fresh buffalo meat in polyamide/polyethylene (PA/PE) packaging during refrigerated storage for 14 days, when treated with cinnamon–clove (C-C) and nutmeg (Nut) powders, along with lavender essential oil (LEO). Microbiological (total viable count, *Pseudomonas* spp., *Brochothrix thermosphacta*, *Enterobacteriaceae*, and lactic acid bacteria), antibacterial (*Salmonella* Typhimurium and *Staphylococcus aureus*), physicochemical and biochemical (pH, moisture, color, total fat, hemoglobin and heme iron, 2-thiobarbituric acid, mercaptans, antioxidant activity, and total phenolic content), and sensory (color, odor, texture, and taste) analyses were carried out. The results showed that C-C and Nut powder extracts exhibited significant (*p* < 0.05) antioxidant and antibacterial activity, higher than LEO; however, all treatments delayed lipid oxidation. Based primarily on sensory evaluation, the shelf life extension of buffalo meat was 2–3 days for LEO and Nut powder, and 4–6 days for C-C powder. Factor analysis indicated the critical days of refrigerated storage for the evolution of spoilage-related biochemical parameters.

## 1. Introduction

In the past decade, there has been a rapid increase in both the consumption and waste of red meat. For this reason, it is necessary to find modern methods to evaluate the degree of meat spoilage and extend its shelf life without chemical food additives. Using spices extracted with edible solvents and essential oils on the surface of the meat is a non-invasive and rapid method [1,2].

Spices and essential oils have been used since ancient times not only to improve the taste of food but also to prevent and treat chronic diseases [3], without any toxic effects being observed [4]. Importantly, they have anti-aging effects, reduce the risk of disease transmission, and possess numerous medicinal properties (antithrombotic, anticancer, antiarrhythmic, etc.).

In the food industry, they have been classified as safe (GRAS) and are used as antimicrobials to inhibit the growth of spoilage microorganisms [5]. Among them, cinnamon and clove consist of a variety of resinous compounds, including cinnamaldehyde (65–75%), cinnamic acid, and many essential oils (trans-cinnamaldehyde, cinnamyl acetate, eugenol, L-borneol, caryophyllene oxide, β-caryophyllene, L-bornyl acetate, E-nerolidol, α-cybene, α-terpenolol, terpinolene, and α-thujene) [6]. Their strong antioxidant activity is attributed to their high content of cinnamaldehyde and eugenol. Their polyphenolic compounds can influence lipid metabolism and prevent oxidation. Cinnamon also contains bioactive compounds that have protective properties against diabetes, obesity, hypertension, and hypercholesterolemia. Cinnamic acids have antioxidant and antihyperglycemic properties and ameliorative effects on diabetic complications [7].

Nutmeg consists mainly of lipids (30–40%) and essential oils (10%). Its characteristic odor is due to the essential oil containing terpenes, terpene derivatives, and phenylpropanes. The antioxidant activity of nutmeg is owed to specific compounds, such as β-caryophyllene and eugenol [7]. On the other hand, lavender and its essential oil comprise an antioxidant agent based on their chemical composition, including lactones and phenolic compounds such as coumarin, herniarin, α-bisabolol, rosmarinic, and chlorogenic acids, and considerable proportions of terpenoids such as caryophyllene oxide (31.8%), spathulenol (10.4%), (E)-caryophyllene (6.9%), 14-hydroxy-9-epi-(E)-caryophyllene (5.3%), elemol (4.9%), and carvacrol (4.4%) [8].

Buffalo meat provides a range of nutrients essential for humans. Proteins account for 95%, consisting of all the essential amino acids, such as leucine and isoleucine, whereas the contained taurine comprises an essential amino acid for newborns, as they cannot synthesize it. Their biological value is estimated at 75 to 100%, and their digestibility is 95% [9]. Importantly, meat from the thigh has less connective tissue but greater biological value [10]. The greater the ratio of the amino acids tryptophan and oxyproline, the greater the biological value of the meat. In buffalo meat, this ratio is 7.2:1, while in cattle it is 5.2:1 [11].

The initial indicator of red meat spoilage is changes in organoleptic characteristics such as unpleasant taste and odor, the appearance of a slimy surface, and undesirable color. The criterion that determines spoilage is when the total microbial load reaches the value of 7 log CFU/g [1]. The type of spoilage differs depending on the microorganism, temperature, and physicochemical characteristics of each meat type [12]. At a constant temperature of 5 °C and with an initial load of 100 CFU/g, 12 days are required until the bacteria grow and reach the spoilage threshold population mentioned previously. The majority of spoilage microflora are Gram-negative bacteria belonging to the genera Pseudomonas, Acinetobacter, Psychrobacter, and Moraxella. These bacteria grow rapidly and displace other microorganisms. Psychrotrophic species of the Enterobacteriaceae family (*Hafnia alvei*, *Serratia liquefancies*, and *Enterobacter agglomerans*) are also part of the microflora of refrigerated meat and are used as meat hygiene indicators. In addition, spoilage is also caused by Gram-positive bacteria such as lactic acid bacteria and *Brochothrix thermosphacta* [13].

Refrigeration has been recognized as the most effective method for preserving meat, combined with either conventional (i.e., polyamide/polyethylene (PA/PE)) packaging [2], biodegradable (i.e., polylactic acid (PLA)) packaging [14], or conventional packaging fortified with antioxidant nanohybrids [15]. After the animal is slaughtered, the enzymes in the meat are highly active, and the bacteria multiply very quickly. Refrigeration slows down the growth of the microbial flora present in the meat as much as possible. Lipid oxidation is the main factor in meat spoilage during its shelf life, causing changes in color, flavor, and aroma. Lipids are oxidized by autoxidation, by the action of the enzyme lipoxygenase, or by photooxidation [16].

On the other hand, hemoglobin also plays a crucial role in initiating the autoxidation of meat lipids, as heme iron has a catalytic effect on the degradation of polyunsaturated fatty acids. Phospholipids are more susceptible to autoxidation phenomena due to their higher content of *ω*-6 linoleic acid [17].

Therefore, the present study aimed to investigate whether the use of selected spice powders (cinnamon and clove mixture, and nutmeg) and essential oils (lavender essential oil) could increase the shelf life of fresh buffalo meat packaged in conventional packaging (PA/PE) under refrigeration. For this purpose, microbiological, antibacterial, physicochemical, biochemical, and sensory analyses were carried out for a period of 14 days. To the best of our knowledge, scarce data regarding the shelf life of fresh buffalo meat are available in the literature, and the special innovation of the present study is that it examines the impact of natural preservatives, such as spice powders and essential oils, to monitor this hypothesis, by collecting microbiological, physicochemical, and sensory analyses data in conjunction with statistical analysis.

## 2. Materials and Methods

### 2.1. Buffalo Meat Samples

The buffalo meat (approximately 4.5 kg, age of 30 months) was received from Kerkini Farm in Serres. Buffalo animals were raised on a free-range pasture diet, with native plants that thrive on Lake Kerkini. The meat was vacuum-packed and shipped to the laboratory. It originated from a male buffalo. After opening the package in the laboratory (day 0), the meat was cut under aseptic conditions into equal proportions of 150 ± 1 g and was packaged into the following packages (treatments): Package A: Control (buffalo meat); Package B: buffalo meat plus cinnamon and clove powder; Package C: Buffalo meat plus nutmeg powder; and Package D: buffalo meat plus lavender essential oil; these packages follow the text sequence. Each package (A–D) consisted of two independent replicates originating from the initial buffalo meat.

### 2.2. Spices and Essential Oils

The spice samples of cinnamon (*Cinnamomum verum*) and clove (*Syzygium aromaticum*) mixture, and nutmeg (*Myristica fragrans*) were purchased from a local store in Agrinio. The samples were ground using a blender to produce a fine powder. The lavender plant (*Lavandula stoechas* L.) originated from the Mani Peninsula (Messenian Gulf), and the essential oil was obtained through hydro-distillation.

### 2.3. Treatments

All treatments studied in the manuscript comprised air-packed atmospheric conditions. The first treatment was packaged directly in a polyamide–polyethylene (PA/PE) package (Control). In the second treatment, cinnamon and clove powder were added to the surface of the meat, at a concentration of 1.5% (*w*/*w*) (this specific concentration used in the treatments, except control samples, was selected based on our previous experience and articles published by our group). It was spread for 5 min to homogenize and cover the entire surface of the meat. Buffalo meat was then packaged in a PA/PE package (C-C). In the third treatment, nutmeg powder was added to the surface of the meat, at a concentration of 1.5% (*w*/*w*). Similarly, it was spread for 5 min to homogenize, and the entire amount was spread quantitatively on the surface of the meat. It was then packaged in a PA/PE package (Nut). Finally, lavender essential oil was added to the surface of the meat at a concentration of 1.5% (*v*/*w*) and treated under the same conditions mentioned above. It was then packaged in a PA/PE package (LEO) (fourth treatment). Finally, all pouches with buffalo meat were stored under refrigeration (4 °C ± 1 °C) for a period of 0, 2, 4, 6, 8, 10, 12, and 14 days.

### 2.4. Packaging Conditions

The buffalo meat samples were placed in PA/PE barrier pouches (29.5 × 29.5 cm, and 90 μm in thickness). The pouches had an oxygen permeability of <15 cm^3^ m^−2^ d^−1^ atm^−1^, a nitrogen permeability of <15 cm^3^ m^−2^ d^−1^ atm^−1^, and a carbon dioxide permeability of <200 cm^3^ m^−2^ d^−1^ atm^−1^ at 75% relative humidity (RH) and 23 °C, in compliance with the standard method DIN 53380-2. In addition, the pouches had a water vapor permeability of <1 g m^−2^ d^−1^ at 85% RH and 23 °C, in compliance with the standard method DIN 53122-2.

### 2.5. Chemicals and Reagents

Ethanol absolute for analysis, trichloroacetic acid (TCA), 2-thiobarbituric acid (TBA), hydrochloric acid, and *n*-hexane were purchased from Merck (Darmstadt, Germany). Acetone was purchased from Centralchem (Gateshead, UK). Starch from potatoes was purchased from Fluka (Seelze, Germany). Iodine and potassium iodide were purchased from Honeywell (Broomfield, CO, USA). Glycerol anhydrous was purchased from Lach-Ner (Neratovice, Czech Republic). 2,2-diphenyl-1-pircylhydrazyl (DPPH) was purchased from Tokyo Chemical Industry Co. (TCI, Tokyo, Japan). Sodium acetate trihydrate was purchased from Uni-chem (Grenville, SC, USA). Folin–Ciocalteu reagent was purchased from Sigma-Aldrich (St. Louis, MO, USA). Finally, gallic acid (3,4,5-trihydrobenzoic acid) 99% isolated from *Rhus chinensis* Mill. was purchased from JNK Tech. Co. (Gunpo-si, Republic of Korea). 

### 2.6. Microbiological Analysis

The evaluation of the microbiological changes in buffalo meat was done by homogenizing 10 g of buffalo meat under each treatment with 90 mL of buffered peptone water (1 g/1000 mL) (BPW, NCM0015A, NEOGEN, Ayr, UK) in a stomacher bag using a Stomacher blender (Interscience, Saint Nom la Breteche, France). Afterward, decimal dilution was carried out using 1 mL of homogenate and 9 mL of peptone water, and then culturing of the obtained dilutions took place on the related agar plates.

For the determination of the total viable count (TVC) of buffalo meat, the surface coating method was used on plate count agar (PCA) substrate (NCM0010A, NEOGEN, Ayr, UK). Approximately 100 μL of the sample was inoculated into each plate using a pipette (Accumax, West Bengal, India), and then the sample was spread on the surface of the substrate using a glass ring. Finally, the plates were stacked upside down and allowed to incubate for 2 days at 30 °C in a digital incubator (Witeg, WIG50, Wertheim, Germany) [2].

For the determination of pseudomonads, the surface coating method was used again on Pseudomonas agar base (CM0559, OXOID, Basingstoke, UK) with the addition of an appropriate amount of glycerol. In brief, 100 μL of the sample was inoculated into each plate using the pipette, and then the sample was spread on the surface of the substrate using the glass ring. Finally, the plates were stacked upside down and allowed to incubate for 2 days at 30 °C in the incubator. The colonies of the pseudomonads were round, smooth, and pale yellow [2].

For the determination of Enterobacteriaceae, the method of incorporation into a selective substrate, Violet Red Bile Glucose Agar (VRBGA, CM0485, OXOID, Basingstoke, UK), was used. For this purpose, 1 mL of the sample was inoculated into each plate using the pipette and then covered with the nutrient substrate. Finally, the plates were stacked upside down and allowed to incubate for 24 h at 37 °C in the incubator (MMM group, ecocell, Planegg, Germany) [2]. The colonies were round and purple.

For the determination of *Brochothrix thermosphacta* in meat, the surface coating method was used on a selective substrate Steptomycin Thallous Acetate Actidione Agar Base (STAA, CM0881, OXOID, Basingstoke, UK) with the addition of an appropriate antibiotic STAA Supplement (4240052, Biolife, Milan, Italy) [1]. In total, 100 μL of the sample was inoculated into each plate using the pipette, and then the sample was spread on the surface of the substrate using a glass ring. Finally, the plates were stacked upside down and allowed to incubate for 2 days at 30 °C in the incubator. The colonies were round, smooth, and intensely beige-yellow in color.

Finally, for the determination of lactic acid bacteria (LAB), the method of incorporation into the selective substrate, de Man Rogosa and Sharpe agar (MRS, NCM0190A, NEOGEN, Ayr, UK), was used. Then, 1 mL of the sample was inoculated into each plate using the pipette and then covered with the nutrient substrate. Finally, the plates were stacked upside down and allowed to incubate for 3 days at 30 °C in the incubator [1]. The colonies were white and lenticular in shape. Microbiological data were transformed into logarithms of the number of colony-forming units (log CFU/g) counted with an ISOLAB (Kalamaria, Greece) counter and presented as average ± standard deviation. Each analysis was carried out on two independent plates.

### 2.7. Antimicrobial Activity

The antimicrobial activity of cinnamon–clove and nutmeg ethanolic extracts, along with lavender essential oil, was tested against two main foodborne pathogenic bacteria from different strains, specifically, the Gram-positive *Staphylococcus aureus* (NCTC 6571) and the Gram-negative *Salmonella* Typhimurium (NCTC 12023). The experimental procedure that followed was based on the method described by Karabagias et al. [14]. Specifically, a final concentration of approximately 1.5 × 10^8^ CFU/mL (0.5 MFU) per bacterial strain was placed in 3 mL of 0.85% peptone salt solution (MRD, Merck). MRD bacteria were inoculated onto Mueller–Hinton agar plates (Oxoid, Basingstoke, UK) using sterile cotton swabs. *Staphylococcus aureus* agar was purchased from CHROMagar (Paris, France). Three 7.3 mm diameter wells were then created under aseptic conditions and inoculated with 150 μL of cinnamon–clove and nutmeg extract, and lavender essential oil, respectively. Two wells were used for each microorganism to ensure the reproducibility of the results. The plates were incubated at 37 °C for 18–24 h in the incubator. The radius of the inhibition zones around the wells was measured using a Vernier caliper to the nearest 0.1 mm.

### 2.8. Determination of Antioxidant Activity

The antioxidant activity of C-C powder extract, Nut powder extract, and LEO extract was determined in vitro by the DPPH radical scavenging assay [18]. For this purpose, two different solvents were used to extract the spice powders and LEO: deionized water (aqueous solvent) and ethanol obtained through the distillation of dry white wine (ethanol of vinous origin, ethanolic solvent) [2]. Results were expressed as percentage inhibition. Reported results are the average ± standard deviation values of three replicates.

### 2.9. Determination of Total Phenolic Content

The total phenolic content of C-C powder extract, nutmeg powder extract, and LEO was determined using the Folin–Ciocalteu colorimetric method [7]. Results were expressed as mg of gallic acid equivalents per liter (mg GAE/L). Reported results are the average ± standard deviation values of three replicates.

### 2.10. Physicochemical Analysis

#### 2.10.1. Determination of pH

For the determination of pH, 20–30 mL of the homogenized and filtered buffalo meat samples in the stomacher bag was transferred to a beaker. The values were measured using a portable pH tester (Hanna, HI98108 pHep+, Athens, Greece) at room temperature, and the results were expressed as pH units at 20 °C. Reported results are the average ± standard deviation values of three replicates.

#### 2.10.2. Determination of Moisture Content

The determination of the moisture content of the meat samples was carried out using the gravimetric drying method [19]. A porcelain capsule was placed in the drying oven at 105 °C for 15 min, and after it had cooled, it was weighed. Then, 5 g of the meat sample, cut into small pieces, was added to it. The capsule was then placed in the drying oven at 105 °C and weighed every half hour until all the moisture was removed and its weight stabilized. Reported results are the average ± standard deviation values of three replicates and expressed as g/100 g.

#### 2.10.3. Determination of Total Fat

To determine the fat content of fresh buffalo meat, 150–200 g of meat was cut into pieces (3–4 g) and placed on sand. The pieces were left in a drying oven at 65 °C for 3 days until the moisture in the meat was completely removed. At the same time, a round-bottomed flask was also dried, allowed to cool in a desiccator, and weighed. Then, the remaining meat from the drying was weighed and separated into three equal parts, which were inserted into cellulose cartridges of the Soxhlet apparatus. Hexane was used as the solvent for the extraction, and the extraction lasted 5 h. Then, the round-bottomed flask with the extract was placed in a drying oven at 65 °C for 2 days, in order to completely evaporate the solvent. Finally, the round flask with the fat was weighed, and the % fat (g/100 g) of the buffalo meat was calculated. Reported results are the average ± standard deviation values of three replicates.

#### 2.10.4. Determination of Color

The color measurement of the samples was performed at the beginning of the physicochemical analyses. The meat samples were first rinsed with deionized water and allowed to dry on filter paper. Then, a colorimeter (LS171, Linshang, Shenzhen, China) was applied to the meat surface. Finally, the results of the parameters L* (lightness), a* (redness when positive, greenness when negative), and b* (yellowness when positive and blueness when negative) were recorded. Reported results are the average ± standard deviation values of three replicates.

#### 2.10.5. Determination of Hemoglobin

The hemoglobin of each treatment was measured after the end of each microbiological analysis using a method developed in our laboratory. From the homogenized solution containing 10 g of meat and 90 mL of buffered peptone water, 20 mL was transferred to a beaker and filtered using Whatman filters. The filtered solution (3 mL) was diluted (1:5) with deionized water and placed in a cuvette, and scans in the range of 400–680 nm were performed with a UV–VIS spectrophotometer (UV-1280, Shimadzu, Kyoto, Japan), to find the maximum absorption of hemoglobin (λ = 413.4 nm). Results were expressed as absorbance units after multiplying the absorbance value by the dilution factor (DF; DF = 6). Reported results are the average ± standard deviation values of three replicates.

#### 2.10.6. Determination of Thiobarbituric Acid Reactive Substances (TBARS)

TBARS were determined according to the recent study of Karabagias et al. [14]. TBARS were calculated in mg of malondialdehyde (MDA)/kg of buffalo meat. Reported results are the average ± standard deviation values of three replicates.

#### 2.10.7. Determination of Heme Iron Content

The heme iron content of buffalo meat was determined according to the method reported by Purohit et al. [20] with slight modifications. Heme iron was expressed as μg/g of buffalo meat. Reported results are the average ± standard deviation values of three replicates.

#### 2.10.8. Determination of Mercaptan Content

The mercaptan content was determined according to Kokkosi et al. [2] with slight modifications. Results were expressed as mg/100 g of buffalo meat. Reported results are the average ± standard deviation values of three replicates.

#### 2.10.9. Sensory Analysis

Eighteen members (Faculty members, PhD candidates, and graduate students experienced in meat analysis) of the Department of Food Science and Technology of the University of Patras carried out the sensory evaluation of the buffalo meat samples. For the sensory analysis, no formal documentation process was available; however, the appropriate protocols for protecting the rights and privacy of all participants were utilized during the execution of the research (i.e., no coercion to participate, full disclosure of study requirements and risks, verbal consent of participants, no release of participant data without their knowledge, and the ability to withdraw from the study at any time).

For each sampling day and each treatment in a monadic test that finished on the same day of sensory evaluation, the color, odor, texture, and taste were evaluated using descriptive tests in a sensory room under controlled lighting. The test samples were cooked in an air fryer (air fryer Izzy IZ-8213, Athens, Greece) at 180 °C for 20 min and then served. Every sample was evaluated individually, and between the different treated samples, panelists rinsed their mouths and cleaned their palates with bottled water. Panelists tested samples that did not exceed the acceptance threshold for total viable count (TVC < 7 log CFU/g). Acceptability of color, odor, texture, and taste was assessed using a 5-to-0 acceptance scale, with 5 representing the most liked sample (highest score value) and 0 representing the least liked sample (lowest score value). A score value of 2.5 was taken as the lowest acceptance threshold [1].

### 2.11. Statistical Analysis

For the statistical processing of the results regarding the effect of applying different preservation methods (treatments) on buffalo meat during storage under refrigeration, one-way analysis of variance (ANOVA) and Tukey’s honestly significant difference (HSD) multiple comparisons test were conducted to assess the significance (*p* < 0.05) of the various parameters. More specifically, in the first case, ANOVA was conducted using the treatments as the factor variable and the measured parameters as the independent variable. In the second case, ANOVA was conducted using the storage time as the factor variable and the measured parameters as the independent variable. Pearson’s correlation (−1 ≤ *r* ≤ +1) bivariate statistics were carried out at the confidence level *p* < 0.05 to find positive or negative correlations between critical parameters among treatments during refrigerated storage. Factor analysis was implemented to indicate the key days of refrigerated storage correlated with the evolution of biochemical parameters of spoilage (TBARS, heme iron, hemoglobin, and mercaptans) [2]. Statistical processing was carried out using the Statistical Package for the Social Sciences (SPSS, v.28.0, IBM Inc., Armonk, NY, USA, 2021).

## 3. Results and Discussion

### 3.1. Antioxidant Activity

The antioxidant activity of C-C, Nut, and LEO extracts is shown in Table 1. Water was selected as a representative solvent, considering that in meat systems, spices and essential oils interact primarily in the presence of water inherent in the tissue. The use of both edible solvents (water and ethanol of vinous origin) aimed to compare their efficiency in extracting antioxidant compounds from C-C and Nut powder extracts, along with LEO, and to evaluate the resulting antioxidant potential.

The antioxidant activity values for both aqueous and ethanolic (vinous-origin) extracts showed statistically significant (*p* < 0.05) differences depending on the type of spice or essential oil used. The highest antioxidant activity was observed in the C-C powder extract, with values of 28.19% for the aqueous and 26.91% for the ethanolic extract at 45 min (plateau of DPPH scavenging). Nut powder extract followed, exhibiting 17.36% (aqueous) and 19.52% (vinous), while LEO recorded the lowest antioxidant activity, with 4.79% and 5.12%, respectively, at 90 min.

Antioxidants play a critical role in a range of biological activities in the human body by preventing the oxidation of other molecules. The antioxidant effect of C-C and Nut can be attributed to the presence of numerous terpenoids and phenolic compounds such as β-caryophyllene, α-pinene, camphene, eugenol, and cinnamaldehyde, which contain hydrogen atoms at allylic or benzylic positions. These positions favor hydrogen donation, making such compounds highly active as antioxidants. The hydrogen atoms are abstracted by peroxyl radicals formed under oxidative stress.

Furthermore, eugenol, present in the used spices, contributes significantly to antioxidant activity by enhancing the activity of specific enzymes, including superoxide dismutase, catalase, glucose-6-phosphate dehydrogenase, glutathione peroxidase, and glutathione transferase [7].

The two solvents demonstrated comparable final antioxidant activity values, with a marginal difference of approximately 1–2%, which was not statistically significant. These findings were further supported by the results of lipid oxidation measurements in the meat samples, where the C-C treatment exhibited the most effective oxidative inhibition, followed by Nut and then LEO.

Similar studies have been conducted on the antioxidant capacity of spices. Lazaridis et al. [7] reported antioxidant activities of 57.24% and 55.12% for 5% ethanolic (vinous-origin) extracts of C-C and Nut powders, respectively.

### 3.2. Total Phenolic Content

As in the case of antioxidant activity, to determine the TPC of spice powders and essential oil, two different solvents were used: deionized water and ethanol of vinous origin (Table 1). The TPC values varied significantly (*p* < 0.05) depending on the spice or essential oil used, for both aqueous and ethanolic extracts. The highest TPC was observed in the C-C extract, with 1048.75 mg GAE/L in the aqueous and 1049.61 mg GAE/L in the ethanolic extract. Nut followed with 215.14 mg GAE/L (aqueous) and 227.57 mg GAE/L (ethanolic), while LEO exhibited the lowest TPC with 3.26 mg GAE/L and 4.29 mg GAE/L, respectively.

Phenolic compounds are known to be primarily responsible for the antioxidant activity of spices and essential oils, confirming a direct correlation between phenolic content and antioxidant performance. The two solvents yielded closely comparable values, with no statistically significant differences in the overall TPC. In line with the antioxidant activity results, C-C extracts ranked highest in both solvents, followed by Nut and LEO extracts.

Major phenolic compounds in cinnamon and clove include eugenol, β-caryophyllene, vanillin, crategolic acid, tannins, gallic acid, methyl salicylate, eugenin, tripentene, oleanolic acid, stigmasterol, and campesterol, alongside several sesquiterpenes. Nutmeg has also been reported to be a rich source of phenylpropanoids such as myristicin, safrole, and elemicin [7]. Similar studies have been conducted on the TPC of spices and essential oils. Lazaridis et al. [7] reported TPC of 1120.24 mg GAE/L and 199.39 mg GAE/L in 5% ethanolic (vinous-origin) extracts of C-C and Nut powders, respectively.

### 3.3. Microbiological Analysis

**Figure 1:**
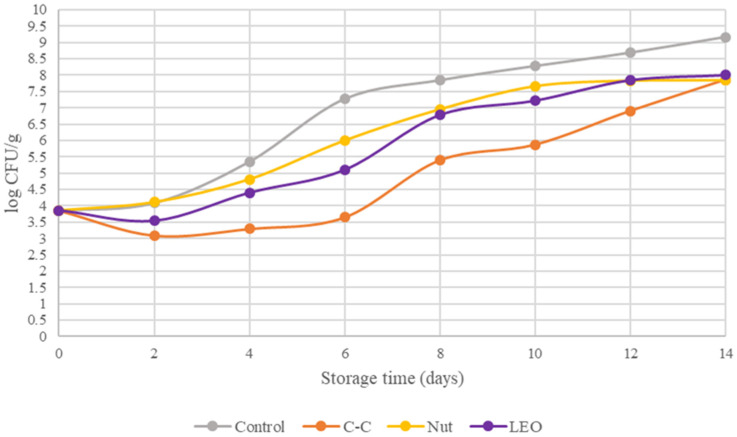
TVC of buffalo meat packaged under different treatments during refrigerated storage.

Figure 1 presents the TVC values for the fresh buffalo meat and the samples supplemented with spices or lavender essential oil. TVC values showed statistically significant (*p* < 0.05) differences during storage under refrigeration. According to previous studies, TVC levels in red meat have been reported to be around 4.7 log CFU/g [21] and 3.55 log CFU/g [22]. In the present study, the TVC on day 0 was 3.86 log CFU/g in the fresh meat, indicating excellent initial microbiological quality. On day 2, the C-C- and LEO-treated samples demonstrated a reduction in the initial microbial load to 3.1 and 3.53 log CFU/g, respectively, compared to the control. The threshold of 7 log CFU/g was reached on days 5–6 in the control samples, on days 12–14 in the C-C samples, and on days 8–10 in the Nut and LEO samples. These results indicate that the addition of spice powders and LEO effectively extended the shelf life of fresh buffalo meat by approximately three to eight days.

**Figure 2:**
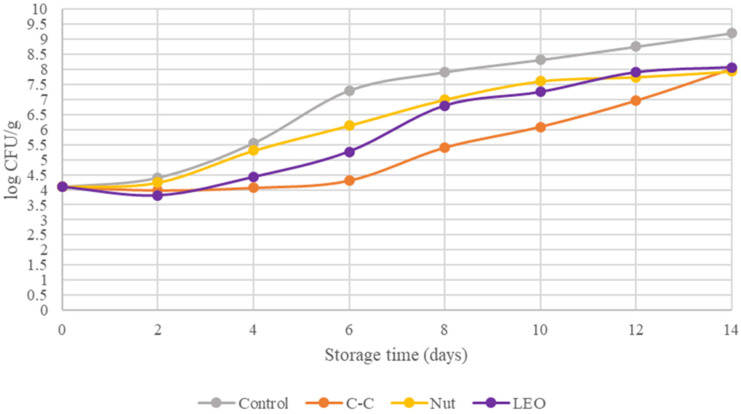
*Pseudomonas* spp. of buffalo meat packaged under different treatments during refrigerated storage.

Among the microbial populations present in buffalo meat, *Pseudomonas* spp. are the predominant spoilage organisms under aerobic storage conditions. The counts of Pseudomonads in the fresh buffalo meat and in the samples treated with spice powders and essential oil are shown in Figure 2. These values demonstrated statistically significant (*p* < 0.05) differences. On day 0, the population of Pseudomonads in control samples was 4.09 log CFU/g. By day 2, the C–C- and LEO-treated samples had reduced Pseudomonas counts to 3.96 and 3.81 log CFU/g, respectively. The spoilage threshold of 7 log CFU/g was reached by Pseudomonads on days 5–6 for the control samples, on days 12–14 for C-C samples, and on days 8–10 for the Nut and LEO samples. Figure 1 and Figure 2 confirm that *Pseudomonas* spp. were the dominant spoilage microorganisms. This is evident from the close correspondence between their growth curves and those of the TVC, a finding that has also been reported by Nychas et al. [23]. Overall, the addition of spice powders and LEO led to an approximate 1.3 log CFU/g reduction in the population of Pseudomonas.

**Figure 3:**
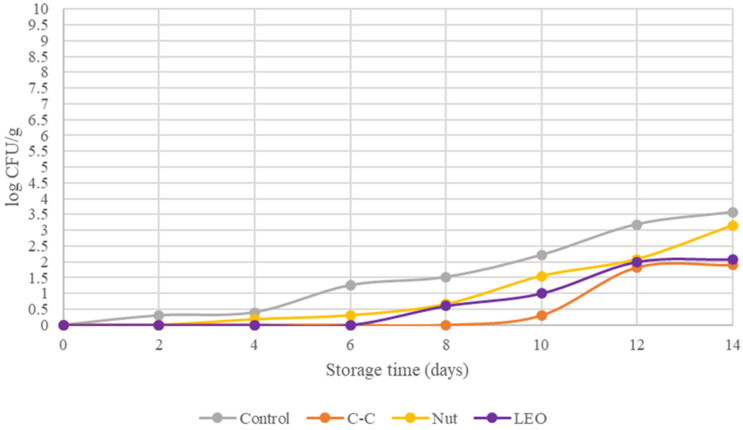
Enterobacteriaceae of buffalo meat packaged under different treatments during refrigerated storage.

The Enterobacteriaceae family, evaluated in the meat samples, is presented in Figure 3. The values of Enterobacteriaceae varied significantly (*p* < 0.05) during refrigerated storage among the treatments. Enterobacteria are considered hygiene indicators in meat, and as facultative anaerobes, they can grow rapidly under vacuum packaging conditions. However, on day 0, the fresh buffalo meat showed no detectable levels of Enterobacteriaceae, indicating excellent meat quality and good hygienic practices during slaughter and processing [24]. Up to days 5–6 of refrigerated storage, the microbial load remained low (<1 log CFU/g), with a slow growth rate. By day 14, the Enterobacteriaceae population reached 3.57 log CFU/g in the control samples. The C-C treatment maintained the Enterobacteriaceae population at undetectable levels until day 8, while LEO inhibited their growth until day 6. By day 14, the counts were 3.15 log CFU/g in the Nut samples, 1.90 log CFU/g in the C-C samples, and 2.07 log CFU/g in the LEO samples. All treatments effectively reduced the initial microbial population. Notably, the C-C and LEO treatments retained Enterobacteriaceae levels within the acceptable limits defined by Regulation (EC) No. 2073/2005 throughout the 14-day refrigerated storage.

**Figure 4:**
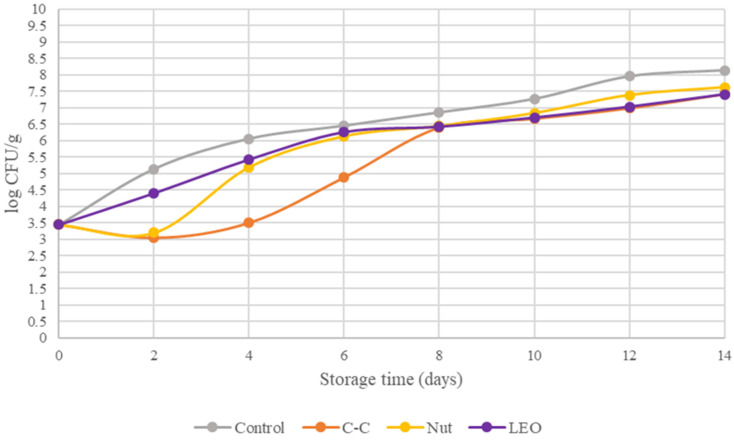
*Brochothrix thermosphacta* of buffalo meat packaged under different treatments during refrigerated storage.

The population of *Brochothrix thermosphacta* in buffalo meat during refrigerated storage is shown in Figure 4. The values exhibit statistically significant (*p* < 0.05) variations. *Brochothrix thermosphacta*, a natural microorganism of the raw meat microbiota, appears to be the second most dominant spoilage microorganism after *Pseudomonas* spp. Its population level closely followed that of Pseudomonas, approximately 1 log CFU/g lower. On day 0, its initial count was 3.44 log CFU/g, which is in agreement with findings reported by Papadopoulou et al. [25]. On day 2, the C-C and Nut samples showed reduced populations of 3.05 and 3.20 log CFU/g, respectively. *Brochothrix thermosphacta* reached 7 log CFU/g on days 8–10 in the control samples, days 12–14 in the C–C samples, and days 10–12 in the Nut and LEO samples. The use of C-C and Nut powders, along with LEO, reduced the initial population of *Brochothrix thermosphacta*.

**Figure 5:**
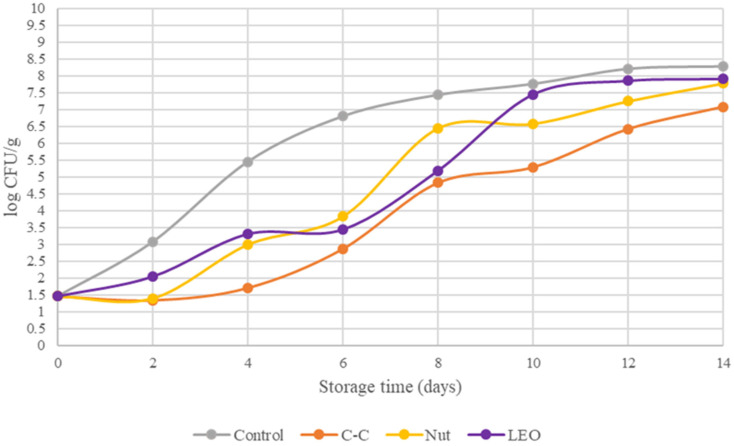
LAB of buffalo meat packaged under different treatments during refrigerated storage.

LAB, along with *Brochothrix thermosphacta*, constitute the secondary microbiota of meat, following *Pseudomonas* spp. in dominance. The LAB values showed statistically significant differences (*p* < 0.05) during refrigerated storage among the treatments (Figure 5). The population of LAB was similar to that of *Brochothrix thermosphacta*. On day 0, their initial population was 1.45 log CFU/g. By day 2 of storage, the C–C and Nut treatments reduced their populations to 1.34 and 1.40 log CFU/g, respectively. LAB reached 7 log CFU/g on days 10–12 in control samples, remained below 7 log CFU/g during the entire storage period in the C–C samples, and reached the threshold of 7 log CFU/g on day 14 in the Nut and LEO treated samples. The use of spice powders and LEO maintained the LAB populations of buffalo meat below 7 log CFU/g during refrigerated storage.

The present findings show that the use of C-C and Nut powders, as well as LEO, effectively reduced the population of the studied microorganisms, thereby extending the shelf life of fresh buffalo meat by approximately 8 days. The C-C powder proved to be the most effective in suppressing microbial growth compared to the Nut powder and LEO. This effect may be attributed to the synergistic action of cinnamaldehyde and eugenol, compounds found in cinnamon and clove, respectively, which are well-documented for their antimicrobial activity [5]. LEO also showed satisfactory results, inhibiting bacterial growth for an additional 2–3 days compared to the control samples. This finding may be attributed to the well-known properties of essential oils, such as the action of bioactive compounds [26], and the reported sensitivity of Gram-positive bacteria to such compounds [27].

In summary, Table 2 shows the average log CFU/g values of the microorganisms studied in relation to each treatment. In bold lettering are the days during which the level of 7 log CFU/g was reached or passed.

Other similar studies have reported shelf life extensions of approximately 2–3 days in lamb meat through the use of 0.1% (*v*/*w*) thyme essential oil [1]. Additionally, an extended shelf life of beef was observed with the use of cinnamon powder [22]. Finally, a reduction in bacterial population and prolonged storage time of lamb meat was also achieved with the addition of cinnamon oil [24].

### 3.4. Antimicrobial Activity

**Figure 6:**
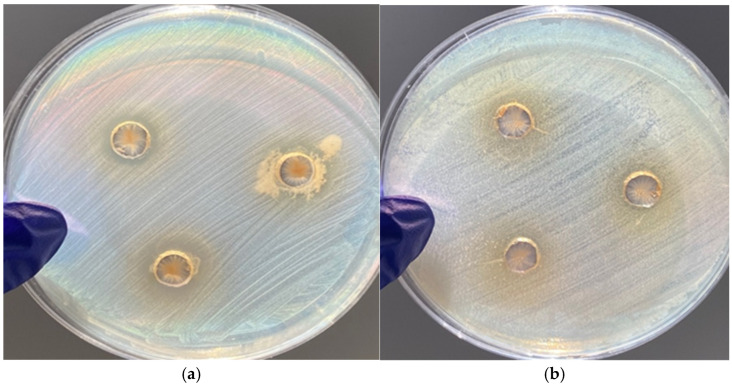
Antimicrobial activity of C-C powder against (**a**) *Staphylococcus aureus* and (**b**) *Salmonella* Typhimurium.

The antimicrobial activity results for Nut powder extract and LEO exhibited no antimicrobial effect against the Gram-positive *Staphylococcus aureus* and Gram-negative *Salmonella* Typhimurium strains. In contrast, the C-C powder extract demonstrated notable antimicrobial activity. Against *Salmonella* Typhimurium, the extract exhibited mild antimicrobial action, inhibiting part of the bacterial growth without complete suppression, and no clear inhibition zone was observed. Against *Staphylococcus aureus*, the C-C powder extract showed strong antimicrobial activity, producing clear inhibition zones with an average diameter of 2.47 ± 0.11 mm (Figure 6). These results indicate that the C-C powder extract has stronger antimicrobial efficacy against Gram-positive bacteria compared to Gram-negative bacteria. This finding is consistent with the results of Nabavi et al. [28], who reported that cinnamon bark extract (*Cinnamomum zeylanicum*) exerted antibacterial effects against methicillin-resistant *Staphylococcus aureus* (MRSA). The cinnamon extract exhibited inhibition zone diameters ranging from 22 to 27 mm and was bactericidal after 6 h of incubation.

### 3.5. Physicochemical Analysis

Table 3 presents the physicochemical and biochemical parameters of buffalo meat among the different treatments during refrigerated storage.

#### 3.5.1. pH

Table 3 shows the pH of buffalo meat and its variations during refrigerated storage. Statistically significant (*p* < 0.05) fluctuations in pH values depending on storage time were observed. pH reflects the degree of meat spoilage and appears to increase in fresh buffalo meat over time. On day 0, the initial pH was 6.38, which is consistent with the literature, while on day 14 of storage, it reached 6.84, showing a steady increase [9]. This increase, as well as the fluctuations observed in all treatments, is justified according to previous studies in the literature [1,21]. It is attributed to the proteolytic activity of the prevailing microorganisms, which produce basic metabolites such as ammonia, free amino acids, sulfides, amines, and low-molecular-weight peptides. Based on the microbiological analysis results, this increase is likely due to the dominant spoilage microorganism, *Pseudomonas* spp. In contrast, any pH decrease is attributed to the activity of microorganisms such as LAB and *Brochothrix thermosphacta*, which produce organic acids during glucose metabolism.

The use of C-C and Nut powders and LEO also influenced pH values, as the final pH value on day 14 of storage was 6.64 for C-C powder and 6.72 for both Nut powder and LEO. This is consistent with the microbiological results, since the C-C powder-treated samples had a lower Pseudomonas population, and thus, the pH increase was less pronounced than in control samples. The same pattern was observed for the other two treatments.

Triki et al. [29] reported that at the end of refrigerated storage, the pH of beef cuts ranged between 6.01 and 7.34, due to the production of nitrogenous basic compounds, mainly amines, which are primary indicators of microbial spoilage. Furthermore, Hussain et al. [22] found that the pH of lamb meat samples treated with cinnamon oil increased significantly over 16 days of storage. In contrast, Karabagias et al. [1] reported a lower pH on day 9 of storage in lamb meat treated with thyme essential oil (0.1%), due to the protective effect of the essential oil against substrate degradation.

#### 3.5.2. Fat Content

The total fat content, determined using the Soxhlet method, showed an average value of 2.82 ± 0.62%. According to Di Stasio et al. [9], buffalo meat is considered a healthier type of red meat, due to its low-fat content, which typically ranges between 1% and 4%. Most of the fat is located subcutaneously, with minimal intramuscular infiltration.

#### 3.5.3. Moisture

Table 3 shows the moisture content of buffalo meat and its variations during refrigerated storage. The reported moisture values showed statistically significant (*p* < 0.05) differences among the different treatments concerning storage time. The fresh buffalo meat initially contained a moisture content of 73.76%. This value is within the typical range for red meat, which contains between 70% and 80% moisture [11], and also aligns with the findings of Maheswarappa et al. [30], who reported that buffalo meat moisture content ranges from 73.04% to 77.75%. During refrigerated storage, the moisture content steadily decreased due to drip loss. By day 14, it had dropped to 66.56%, corresponding to a total moisture loss of 7.2%.

The samples treated with C-C powder maintained a more stable moisture content, declining from 73.76% to 71.08% by day 14, resulting in a moisture loss of only 2.68%. The powder of C-C appears to act as a surface barrier, sealing the meat tissue and preventing moisture loss. Even though a small amount of moisture is lost during refrigerated storage, the spice powders and LEO absorbed moisture, preventing the accumulation of drip inside the packaging, which could otherwise become a source of contamination. This observation is further supported by the microbiological analysis results we collected, as moisture accumulation inside the packaging could promote extensive microbial growth.

Nut powder showed similarly satisfactory performance, resulting in a 3.7% moisture loss and a final moisture content of 70.06% on day 14. Lastly, LEO initially increased the meat’s moisture content, likely due to its liquid nature, but later allowed for a moisture loss of 4.98%, reaching 68.78% on day 14. Both spices and LEO proved to be effective in preserving the initial moisture content and reducing drip loss.

#### 3.5.4. Color

The results regarding the color changes of buffalo meat during refrigerated storage are shown in Table 3. Maintaining an appealing color is of great importance, as color is the first sensory criterion that consumers use to evaluate meat quality. The L* values, which represent lightness, generally decreased significantly (*p* < 0.05) during refrigerated storage in all treatments. The a* values, which indicate redness, decreased in fresh buffalo meat, whereas in the other treatments they showed fluctuations during storage time. The decrease in a* values is attributed to myoglobin oxidation and the formation of metmyoglobin. For the b* values, which relate to yellowness, similar fluctuations were observed during refrigerated storage. An increase in the b* parameter may be associated with the formation of metmyoglobin, which occurs more rapidly at relatively low O_2_ concentrations.

Overall, despite the observed fluctuations, the samples treated with spices and essential oil maintained more stable color characteristics compared to fresh buffalo meat, in agreement with previous studies in the literature [1].

#### 3.5.5. Hemoglobin

Hemoglobin (Hb) is a protein responsible for oxygen transport in mammals. It assists in the transport and storage of oxygen and is involved in redox reactions. Statistically significant (*p* < 0.05) variations in hemoglobin absorbance values were observed among treatments during refrigerated storage (Table 3). Since hemoglobin contains bound iron in its structure, it is directly linked to heme iron and its variations. Fresh buffalo meat initially showed a hemoglobin absorbance of 5.06. During refrigerated storage, this value was decreased, which can be attributed to drip loss, as the iron from hemoglobin is lost within the exudate. Additionally, this reduction may result either from the oxidation of iron during refrigerated storage from Fe^2+^ to Fe^3+^ (a non-bioavailable form) or from microbial metabolic activity that produces metabolites that accelerate iron oxidation. On the last day of buffalo meat storage, hemoglobin absorbance dropped to 1.86. A similar trend was observed in the samples treated with Nut powder and LEO, where absorbance values decreased from 5.06 to 2.57 and 2.23, respectively, on day 14. In contrast, the C-C powder appeared to be more effective, maintaining a higher absorbance value of 3.38 on day 14. This improved performance is probably due to its dual action: preventing drip loss, as previously discussed, and exhibiting strong antioxidant properties. There was a strong positive and significant (*p* < 0.001) Pearson’s correlation between heme iron content and hemoglobin values among treatments during refrigerated storage (Table 4).

**Figure 7:**
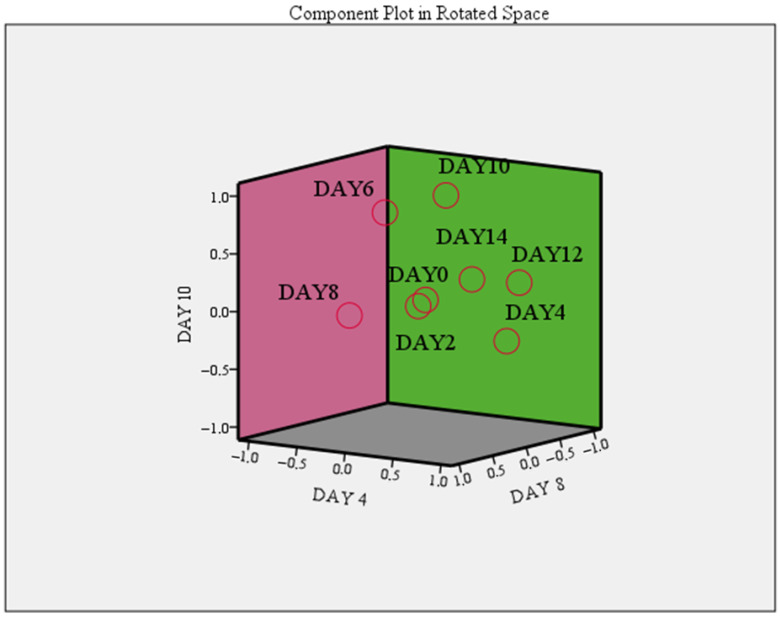
Refrigerated storage as a critical parameter for hemoglobin evolution.

To date, no other studies have reported measurements of hemoglobin absorbance values in red meat or buffalo meat treated with spices and essential oils during refrigerated storage. These findings regarding hemoglobin content are consistent with the results observed for heme iron and its variation during refrigerated storage (see the next subsection). Factor analysis indicated that the principal components were related to the evolution of hemoglobin. These were day 4 (principal component 1, PC1), day 10 (principal component 2, PC2), day 8 (principal component 3, PC3), and day 0 (principal component 4, PC4) of refrigerated storage, which explained 99.82% of the total variance. The highest variance was explained by PC1 (36.87%, correlation of 0.976 in the rotated space), followed by PC2 (25.27%, correlation of 0.971 in the rotated space), PC3 (25.15%, correlation of 0.997 in the rotated space), and PC4 (12.53%, correlation of 1.000 in the rotated space) (Figure 7).

#### 3.5.6. TBARS

Lipid oxidation is one of the most significant and inevitable chemical alterations in meat. When meat comes into contact with oxygen, the unsaturated fatty acids in the adipose tissue react with oxygen and form compounds such as aldehydes and ketones, which contribute to unpleasant odor and taste. The TBARS values for fresh buffalo meat are shown in Table 3, showing statistically significant (*p* < 0.05) differences among treatments during refrigerated storage.

All TBARS values for the buffalo meat ranged between 0.38 and 1.06 mg MDA/kg, indicating a very low level of lipid oxidation. Fresh buffalo meat initially exhibited a TBARS value of 0.38 mg MDA/kg, which increased to 0.87 mg MDA/kg on day 14 of refrigerated storage. Karabagias et al. [1] reported TBARS values ranging from 1.4 to 3.8 mg MDA/kg in fresh lamb meat after 9 days of storage under aerobic conditions. Similarly, Karabagias et al. [14] reported values between 0.46 and 0.81 mg MDA/kg for pork stored for 6 days using PLA-based packaging.

The C-C powder proved to be particularly effective in maintaining TBARS values low, increasing from 0.38 on day 0 to only 0.65 mg MDA/kg meat on day 14. This is attributed to the high content of phenolic compounds in these spices, which are known for their strong antioxidant activity. Cinnamaldehyde and eugenol are capable of affecting lipid metabolism and preventing lipid oxidation [7]. This is further supported by the in vitro antioxidant activity and total phenolic measurements of the spice extracts used in the present study (see Section 3.1 and Section 3.2).

Similarly, Nut powder also contributed to controlling lipid oxidation, with TBARS values rising from 0.38 on day 0 to 0.77 mg MDA/kg on day 14, given its considerable antioxidant activity and total phenolic content (see Section 3.1 and Section 3.2). The antioxidant effect of nutmeg is due to various compounds it contains, including, among others, β-caryophyllene and eugenol. On the other hand, LEO did not protect buffalo meat from lipid oxidation as efficiently as the other treatments. This finding is consistent with the results of Karabagias et al. [1], who reported that thyme essential oil at 0.1% (*v*/*w*) failed to inhibit lipid oxidation in lamb meat.

**Figure 8:**
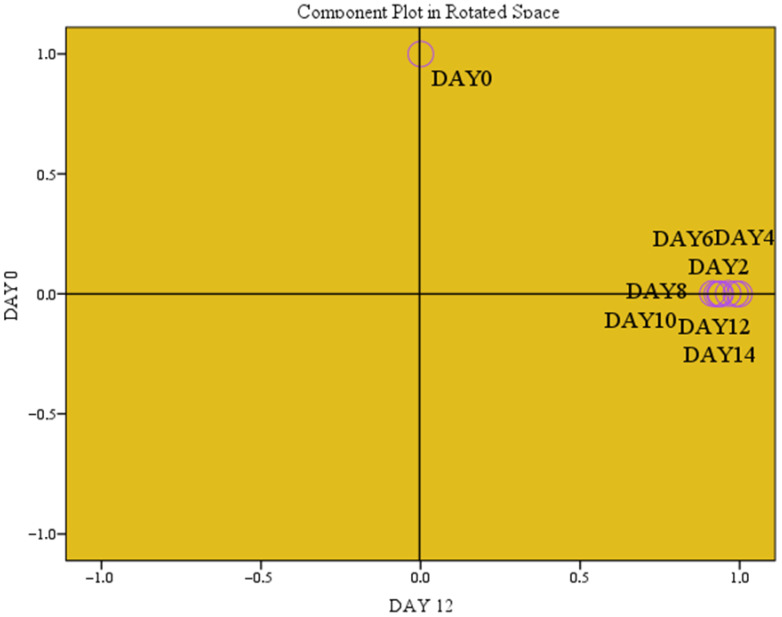
Refrigerated storage as a critical parameter on TBARS evolution.

There are no regulatory limits for MDA content in meat. However, values exceeding 0.5 mg MDA/kg suggest some degree of oxidation, while levels above 1.0 mg/kg are considered potentially undesirable [31]. Lipid oxidation has been associated with rancid odor and taste in stored meat products, with TBARS values ≥5 mg MDA/kg considered the threshold for detectable rancidity [1]. Such high values were not observed in the present study. Factor analysis indicated the principal components related to the evolution of TBARS. These were day 12 (principal component 1, PC1) and day 0 (principal component 2, PC2) of refrigerated storage, which explained 92.04% of the total variance. The highest variance was explained by PC1 (79.54%, correlation of 0.999 in the rotated space), followed by PC2 (12.50%, correlation of 1.000 in the rotated space) (Figure 8).

#### 3.5.7. Heme Iron

The high heme iron content in buffalo meat is what gives it significant nutritional value. Initially, the heme iron content was 19.13 μg/g of meat. According to Di Stasio et al. [9], Tamburrano et al. [32], and Maheswarappa et al. [30], the heme iron content in buffalo meat typically ranges between 14 and 26 μg/g of meat. The heme iron content values showed statistically significant (*p* < 0.05) variations among treatments during refrigerated storage (Table 3). Throughout the storage period, a downward trend in heme iron content was observed, decreasing from 19.13 μg/g on day 1 to 10.95 μg/g on day 14. This decline was expected, as iron is oxidized during storage from Fe^2+^ to Fe^3+^, the latter being a non-bioavailable form, leading to nutritional losses. Additionally, the loss of drip during storage also results in the loss of hemoglobin-bound iron. The meat samples treated with the C-C and Nut powder performed satisfactorily, maintaining heme iron content at 14.39 and 12.62 μg/g, respectively, on day 14. This preservation effect is probably due to the spices’ ability to inhibit oxidation and form a coating on the surface of the meat that protects tissue integrity and minimizes drip loss. Furthermore, certain microorganisms that grow during refrigerated storage may influence iron content either by directly consuming iron or by producing metabolites that accelerate its oxidation. This finding is also supported by microbiological analysis, which showed that the C-C powder significantly reduced the microbial load of buffalo meat. There was a strong positive and significant (*p* < 0.001) Pearson’s correlation between heme iron content and TVC values among treatments during refrigerated storage (Table 5).

**Figure 9:**
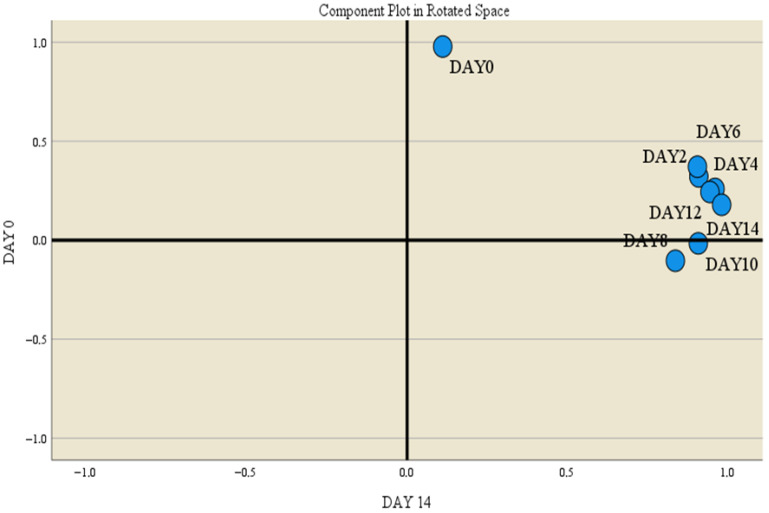
Refrigerated storage as a critical parameter on heme iron evolution.

On the contrary, LEO appeared to be less effective in preserving heme iron content, as the content dropped to 9.60 μg/g on day 14. These results are in agreement with the hemoglobin absorbance values, since hemoglobin contains bound iron that is similarly lost during storage. Overall, the spice powders and particularly the C-C powder are ideal for preventing iron loss and preserving heme iron at high levels. From a nutritional perspective, this is significant for human health, as it enhances the nutritional value of meat. Factor analysis indicated the principal components related to the evolution of heme iron. These were day 14 (principal component 1, PC1) and day 0 (principal component 2, PC2) of refrigerated storage, which explained 91.94% of the total variance. The highest variance was explained by PC1 (74.82%, correlation of 0.983 in the rotated space), followed by PC2 (17.12%, correlation of 0.979 in the rotated space) (Figure 9).

#### 3.5.8. Mercaptans

Table 3 shows the concentration of mercaptans in buffalo meat and their variation during refrigerated storage. Mercaptans are formed by the progressive degradation of sulfur-containing amino acids, a process driven by certain microorganisms, resulting in the production of hydrogen sulfide (H_2_S). Their presence reflects the degree of meat spoilage, as they significantly influence its organoleptic properties [33].

Mercaptan content on days 2, 4, and 6 did not vary significantly (*p* > 0.05) by the use of spices or essential oil. However, from day 8 onwards (days 8, 10, 12, and 14), statistically significant (*p* < 0.05) differences were observed among treatments during refrigerated storage. Initially, fresh buffalo meat had a mercaptan content of 214.40 mg/100 g. Liang et al. [33] reported an initial concentration of 95.0 mg/100 g in lamb meat stored at 0 °C.

A continuous increase in mercaptan content was observed throughout refrigerated storage, primarily due to microbial metabolic activity. The bacteria responsible include *Weissella viridescens*, *Leuconostoc* spp., *Enterococcus faecium*, *Enterococcus faecalis*, and members of the Enterobacteriaceae family. Based on the microbiological analysis results, since the Enterobacteriaceae population remained particularly low, the increase in mercaptan content is likely attributed to other Gram-positive bacteria, such as LAB. There was a strong positive and significant (*p* < 0.001) Pearson’s correlation between mercaptan content and LAB population among treatments during refrigerated storage (Table 6).

By day 14, control buffalo meat samples reached a mercaptan content of 920.02 mg/100 g. Among the treatments, Nut powder and especially the C-C powder maintained significantly (*p* < 0.05) lower levels of mercaptans, likely due to their antimicrobial effects, which in turn reduced bacterial load. Specifically, on day 14, the C-C and Nut powders reached mercaptan content equal to 619.73 and 870.20 mg/100 g, respectively. In contrast, the sample treated with LEO showed a similar trend to the control samples and slightly surpassed it, reaching ca. 1011 mg/100 g.

**Figure 10:**
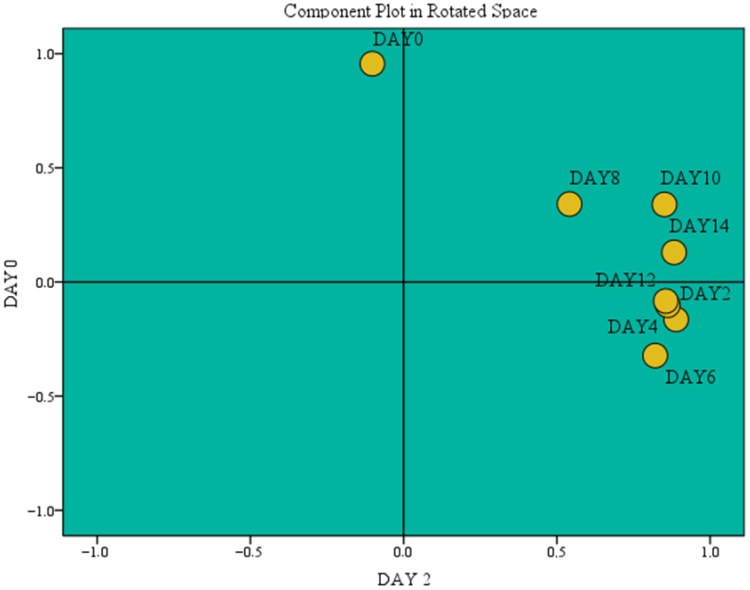
Refrigerated storage as a critical parameter on mercaptan evolution.

Despite the relatively satisfactory microbiological profile of the LEO-treated meat, it did not appear effective in reducing mercaptan formation. This suggests that LEO may not significantly inhibit specific microbial pathways mentioned previously (i.e., LAB), responsible for the production of sulfur compounds. However, this is a finding that requires additional research. Factor analysis indicated the principal components related to the evolution of mercaptans. These were day 2 (principal component 1, PC1) and day 0 (principal component 2, PC2) of refrigerated storage, which explained 75.74% of the total variance. The highest variance was explained by PC1 (59.35%, correlation of 0.889 in the rotated space), followed by PC2 (16.40%, correlation of 0.956 in the rotated space) (Figure 10).

#### 3.5.9. Sensory Analysis

**Figure 11:**
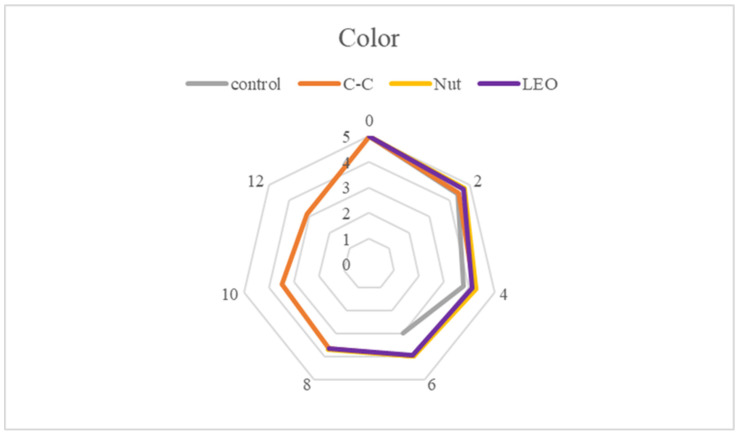
Color scores of buffalo meat packaged under different treatments during refrigerated storage.

**Figure 12:**
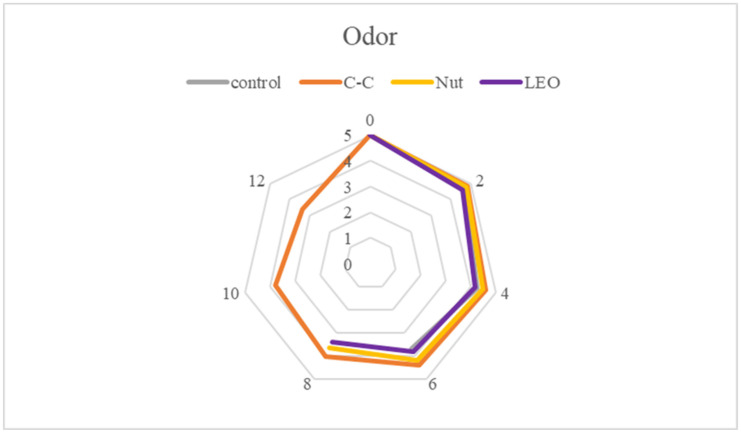
Odor scores of buffalo meat packaged under different treatments during refrigerated storage.

**Figure 13:**
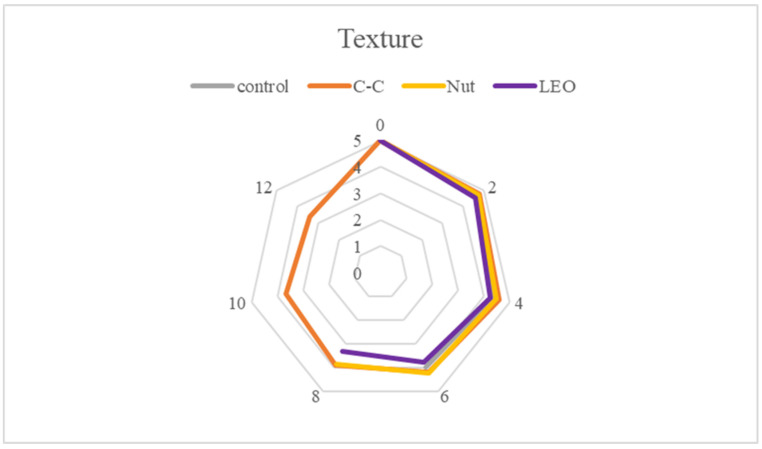
Texture scores of buffalo meat packaged under different treatments during refrigerated storage.

**Figure 14:**
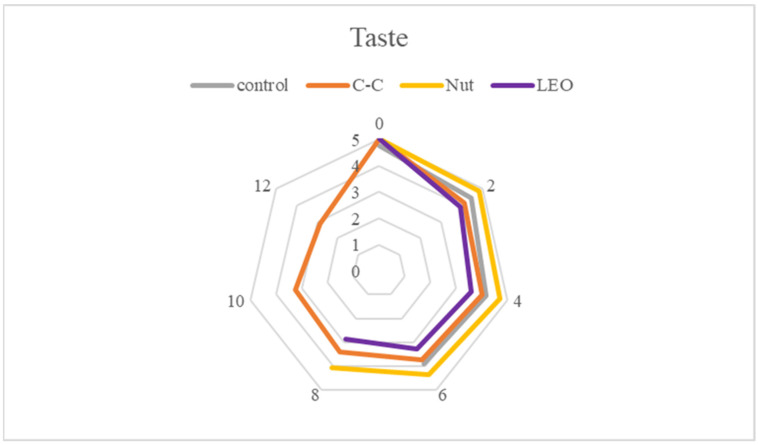
Taste scores of buffalo meat packaged under different treatments during refrigerated storage.

Sensory evaluation aimed to assess the organoleptic characteristics of buffalo meat, focusing on color (appearance), odor, texture (firmness), and taste (Figure 11, Figure 12, Figure 13 and Figure 14). Each sample was presented in randomized order and coded to ensure unbiased evaluation. Panelists used a structured hedonic scale to score each parameter, where higher values indicated greater acceptability. The assessments were conducted multiple times during the storage period to capture potential variations over time. Samples with mean sensory scores below 2.5 were considered unacceptable and unsuitable for consumption. In the present study, all values recorded were above 2.88, indicating that, apart from being microbiologically acceptable, the samples were also organoleptically acceptable throughout refrigerated storage.

Statistically significant (*p* < 0.05) differences were observed in color (Figure 11) and odor (Figure 12) scores depending on storage time. However, the color score on day 8 and the odor scores on days 2 and 4 were not significantly (*p* > 0.05) affected by the addition of spices or essential oil. The intense red color and characteristic odor of fresh buffalo meat began to deteriorate between days 4 and 6, as confirmed by lipid oxidation levels and microbiological analyses. From day 4 onward, the TVC was between 5 and 7 log CFU/g, while MDA levels exceeded 0.5 mg/kg, indicating the development of microbial flora and the production of spoilage-related metabolites responsible for unpleasant odors. Additionally, the oxidation of myoglobin led to a color shift toward brown.

From a sensory perspective, nutmeg was effective in masking the unpleasant odor that developed and in preserving the color of the meat throughout refrigerated storage. The C-C treatment imparted a characteristic aroma that covered spoilage odors, but gave the meat a darker appearance, which confused some panelists and was perceived as less attractive. The LEO treatment exhibited a similar sensory degradation pattern to the control. Although color was slightly improved, the combination of spoilage odor and lavender volatiles was not considered pleasant by several panelists.

Meat tenderness is one of the most important quality attributes for consumers, as it reflects both the perceived softness and the cohesion of muscle fibers. Statistically significant (*p* < 0.05) differences in texture scores (Figure 13) were observed depending on storage time. However, on days 2 and 4, no significant (*p* > 0.05) differences were observed among treatments. Control buffalo meat samples maintained acceptable texture up to day 6. Among the treatments, C-C and Nut powders preserved better overall texture compared to the control samples, likely due to their ability to retain moisture and juiciness within the meat tissues. Nevertheless, some samples developed a sticky surface (“slime”) on days with higher TVC, which was noted negatively by panelists.

As for the taste of air-fried buffalo meat, scores varied among treatments due to subjective consumer preferences (Figure 14). However, significant (*p* < 0.05) differences were observed depending on storage time and treatment. Control buffalo meat samples did not show intense flavor deterioration during storage and were characterized by panelists as a high-quality red meat with distinctive sensory attributes suitable for incorporation into the Mediterranean diet. According to Karabagias et al. [1], the off-flavor in aerobically packaged meats is mainly attributed to volatile metabolites produced by extensive populations of *Pseudomonas* spp.

Nut powder preserved the original flavor of the meat and enhanced its overall sensory quality with a subtle and complementary aroma. In contrast, the C-C powder, at the tested concentration, masked the meat’s natural flavor and left a slightly bitter aftertaste due to its volatile phenolic compounds. The LEO was deemed an unsuitable flavoring agent, as its strong phenolic notes were perceived as incompatible with the meat’s flavor profile during mastication.

Based on sensory evaluation, the shelf life of fresh buffalo meat can be extended by approximately 2–8 days with the addition of C-C and Nut powders, along with LEO. Although the C-C treatment demonstrated the most promising results in microbiological and physicochemical analyses, effectively doubling the shelf life, the Nut treatment proved to be the most suitable sensory treatment according to panelists. Karabagias et al. [1] reported that the shelf life of fresh lamb meat based on sensory analysis can be extended by 2–3 days with the addition of 0.1% (*v*/*w*) thyme essential oil, which was also well-accepted by panelists.

## 4. Conclusions and Future Perspectives

The results of the present study showed that C-C and Nut powders, along with LEO, added on the surface of buffalo meat packaged in PA/PE during refrigeration, could increase its shelf life. C-C powder proved to be more effective in suppressing microbial growth, compared to Nut powder and LEO. Both spices and LEO also decreased lipid oxidation and the formation of off odor (mercaptans). The C-C aqueous and ethanolic extracts were the only ones that had antimicrobial activity against the pathogenic bacteria *Salmonella* Typhimurium and *Staphylococcus aureus*. The results of sensory evaluation are in general agreement with those of microbiological and physicochemical analyses. Although C-C powder had a better preservation effect, the panelists preferred Nut powder. Nut powder managed to keep the color of meat stable during refrigeration, to cover unpleasant odors, and to enhance the aromas of buffalo meat during air frying. Based primarily on sensory evaluation, the shelf life of buffalo meat was 4–6 days for the control samples, 6–9 days for LEO- and Nut-treated samples, and 10–12 days for C-C-treated samples. To the best of our knowledge, scarce data is available in the literature with the current hypothesis, correlating microbiological analysis data of buffalo meat with biochemical parameters (i.e., TVC with heme iron and mercaptans with LAB), and in parallel highlighting that the storage time of meat affects differently the biochemical pathways related to the development of hemoglobin, heme iron, TBARS and mercaptans, as shown by factor analysis. These data constitute the novelty of the present research study. However, for potential application of the present data in the food industry, and in particular in the meat industry, there might be some modifications owed mainly to the amount of the specific natural preservatives used, considering mainly the cost of application and any regulatory constraints.

## Figures and Tables

**Table 1:** Antioxidant activity and total phenolic content of aqueous and ethanolic extracts of spice powders and LEO.

Extracts	Antioxidant Activity (%)	Antioxidant Activity (%)	TPC (mg GAE/L)	TPC (mg GAE/L)
	Water	Ethanol	Water	Ethanol
C-C	28.19 ± 1.05 ^aA^	26.91 ± 1.36 ^aA^	1048.75 ± 8.96 ^aA^	1049.61 ± 8.32 ^aA^
Nut	17.36 ± 2.73 ^aB^	19.52 ± 2.73 ^aB^	215.14 ± 1.69 ^aB^	227.57 ± 2.30 ^aB^
LEO	4.79 ± 0.32 ^aC^	5.12 ± 0.52 ^aC^	3.26 ± 1.59 ^aC^	4.80 ± 2.56 ^aC^

C-C: cinnamon and clove powder. Nut: Nutmeg powder. LEO: Lavender essential oil. TPC: Total phenolic content. The same lower-case letters in each row indicate non-statistically significant (*p* > 0.05) differences. The different upper-case letters in each column indicate statistically significant (*p* < 0.05) differences.

**Table 2:** Summary table of the average values of the population of microorganisms (log CFU/g) of buffalo meat during refrigerated storage in relation to each treatment.

	**TVC**							
	Day 0	Day 2	Day 4	Day 6	Day 8	Day 10	Day 12	Day 14
Control	3.86	4.09	5.36	**7.27**	7.84	8.28	8.68	9.16
C-C	3.86	3.09	3.30	3.65	5.40	5.87	6.90	7.86
Nut	3.86	4.11	4.81	5.99	**6.95**	7.66	7.83	7.84
LEO	3.86	3.52	4.40	5.10	6.78	**7.22**	7.84	7.50
	***Pseudomonas*** **spp.**							
	Day 0	Day 2	Day 4	Day 6	Day 8	Day 10	Day 12	Day 14
Control	4.09	4.40	5.55	**7.30**	7.92	8.32	8.76	9.21
C-C	4.09	3.96	4.06	4.30	5.40	6.09	**6.95**	8.00
Nut	4.09	4.24	5.30	6.13	**6.99**	7.60	7.74	7.93
LEO	4.09	3.81	4.42	5.27	6.79	**7.25**	7.90	8.07
	**Enterobacteriaceae**							
	Day 0	Day 2	Day 4	Day 6	Day 8	Day 10	Day 12	Day 14
Control	0.00	0.30	0.39	1.25	1.51	2.20	3.18	3.57
C-C	0.00	0.00	0.00	0.00	0.15	0.24	1.83	1.90
Nut	0.00	0.15	0.15	0.30	0.57	1.54	2.07	3.15
LEO	0.00	0.00	0.00	0.15	0.59	0.98	2.00	2.07
***Brochothrix thermosphacta***								
	Day 0	Day 2	Day 4	Day 6	Day 8	Day 10	Day 12	Day 14
Control	3.44	5.14	6.06	6.46	6.86	**7.27**	7.96	8.14
C-C	3.44	3.05	3.50	4.89	6.40	6.67	**6.99**	7.42
Nut	3.44	3.20	5.20	6.14	6.45	6.84	**7.39**	7.62
LEO	3.44	4.41	5.43	6.26	6.43	6.70	**7.04**	7.41
**LAB**								
	Day 0	Day 2	Day 4	Day 6	Day 8	Day 10	Day 12	Day 14
Control	1.45	3.08	5.45	5.80	6.44	6.76	**7.21**	7.28
C-C	1.45	1.34	1.70	2.86	3.83	4.29	5.42	6.08
Nut	1.45	1.40	2.98	3.82	5.45	5.56	6.25	6.78
LEO	1.45	2.05	3.31	3.45	4.19	6.45	6.86	6.91

**Table 3:** Physicochemical and biochemical parameters of buffalo meat among the different treatments during refrigerated storage.

Control	Day 0	Day 2	Day 4	Day 6	Day 8	Day 10	Day 12	Day 14
pH	6.38 ± 0.01 ^Aa^	6.50 ± 0.01 ^Ab^	6.51 ± 0.01 ^Ab^	6.55 ± 0.01 ^Ac^	6.58 ± 0.01 ^Ad^	6.63 ± 0.01 ^Ae^	6.75 ± 0.01 ^Af^	6.84 ± 0.01 ^Ag^
Moisture (g/100 g)	73.76 ± 1.04 ^Aa^	71.64 ± 0.83 ^Aa^	69.83 ± 0.96 ^Aab^	68.14 ± 0.91 ^Ab^	67.63 ± 1.29 ^Ab^	67.12 ± 0.78 ^Ab^	66.78 ± 1.06 ^Abc^	66.56 ± 1.03 ^Abc^
L*	43.23 ± 0.47 ^Aa^	41.56 ± 0.28 ^Ab^	38.04 ± 0.87 ^Ac^	36.36 ± 0.41 ^Ad^	35.50 ± 0.31 ^Ad^	32.24 ± 0.37 ^Ae^	30.69 ± 0.33 ^Af^	28.73 ± 0.87 ^Ag^
a*	6.76 ± 0.98 ^Aa^	7.64 ± 0.66 ^Aa^	6.96 ± 0.67 ^Aa^	5.80 ± 0.38 ^Aa^	5.45 ± 1.33 ^Aab^	4.25 ± 0.46 ^Aab^	3.45 ± 0.30 ^Ab^	2.94 ± 0.27 ^Ab^
b*	6.52 ± 0.32 ^Aa^	5.17 ± 0.07 ^Aa^	5.45 ± 1.34 ^Aa^	3.47 ± 0.92 ^Aab^	2.91 ± 0.50 ^Aab^	3.55 ± 1.31 ^Aab^	2.07 ± 0.84 ^Ab^	5.17 ± 0.07 ^Aab^
Heme iron (μg/g)	19.13 ± 0.01 ^Aa^	17.69 ± 0.01 ^Ab^	15.89 ± 0.01 ^Ac^	15.68 ± 0.01 ^Ad^	13.19 ± 0.01 ^Ae^	13.16 ± 0.01 ^Af^	12.62 ± 0.01 ^Ag^	10.95 ± 0.01 ^Ah^
Hemoglobin (AU)	5.06 ± 0.01 ^Aa^	4.29 ± 0.01 ^Ab^	3.97 ± 0.01 ^Ac^	4.31 ± 0.01 ^Ab^	3.70 ± 0.01 ^Ad^	3.99 ± 0.01 ^Ac^	2.06 ± 0.01 ^Ae^	1.86 ± 0.01 ^Af^
TBARS (mg MDA/kg)	0.38 ± 0.00 ^Aa^	0.46 ± 0.01 ^Ab^	0.57 ± 0.02 ^Ac^	0.70 ± 0.02 ^Ad^	0.63 ± 0.03 ^Ae^	0.73 ± 0.03 ^Af^	0.82 ± 0.03 ^Ag^	0.87 ± 0.01 ^Ah^
Mercaptans (mg/100 g)	214.40 ± 0.79 ^Aa^	329.07 ± 41.23 ^Ab^	412.74 ± 41.72 ^Ab^	498.28 ± 45.18 ^Abc^	664.94 ± 37.82 ^Ad^	647.45 ± 43.00 ^Ad^	839.34 ± 41.14 ^Ae^	920.02 ± 41.29 ^Ae^
**C-C**	Day 0	Day 2	Day 4	Day 6	Day 8	Day 10	Day 12	Day 14
pH	6.38 ± 0.01 ^Aa^	6.44 ± 0.01 ^Bb^	6.52 ± 0.01 ^Ac^	6.54 ± 0.01 ^Ad^	6.62 ± 0.01 ^Be^	6.55 ± 0.01 ^Bd^	6.54 ± 0.01 ^Bcd^	6.64 ± 0.01 ^Bf^
Moisture (g/100 g)	73.76 ± 1.04 ^Aa^	72.30 ± 0.89 ^Aa^	72.22 ± 1.04 ^ABa^	72.15 ± 0.64 ^Bb^	71.83 ± 1.28 ^Ba^	71.66 ± 0.89 ^Ba^	71.27 ± 0.72 ^Ba^	71.08 ± 1.18 ^Ba^
L*	43.23 ± 0.47 ^Aa^	38.36 ± 0.49 ^Bb^	36.60 ± 0.44 ^Ac^	36.36 ± 0.53 ^Ac^	35.70 ± 0.56 ^Ac^	35.70 ± 0.03 ^Bc^	30.88 ± 0.70 ^Ad^	29.09 ± 0.54 ^Ae^
a*	6.76 ± 0.98 ^Aa^	7.64 ± 0.66 ^Aa^	9.11 ± 0.65 ^Ab^	6.32 ± 0.27 ^Aa^	6.12 ± 0.28 ^Aa^	9.11 ± 0.07 ^Bb^	10.08 ± 0.24 ^Bb^	6.47 ± 0.16 ^Ba^
b*	6.52 ± 0.32 ^Aa^	5.47 ± 0.31 ^Ab^	6.89 ± 0.10 ^Aa^	4.22 ± 0.08 ^Ac^	4.78 ± 0.39 ^Ac^	8.91 ± 0.04 ^Bd^	5.08 ± 0.09 ^Bb^	5.68 ± 0.02 ^Ab^
Heme iron (μg/g)	19.13 ± 0.01 ^Aa^	19.04 ± 0.01 ^Bb^	18.44 ± 0.01 ^Bc^	18.89 ± 0.01 ^Bd^	16.88 ± 0.01 ^Be^	15.32 ± 0.01 ^Bf^	15.05 ± 0.01 ^Bg^	14.39 ± 0.01 ^Bh^
Hemoglobin (AU)	5.06 ± 0.01 ^Aa^	4.96 ± 0.01 ^Bb^	4.65 ± 0.01 ^Bc^	4.68 ± 0.01 ^Bc^	4.16 ± 0.01 ^Bd^	4.24 ± 0.01 ^Be^	4.01 ± 0.01 ^Bf^	3.38 ± 0.01 ^Bg^
TBARS (mg MDA/kg)	0.38 ± 0.00 ^Aa^	0.39 ± 0.01 ^Bb^	0.45 ± 0.02 ^Bc^	0.46 ± 0.01 ^Bd^	0.50 ± 0.01 ^Be^	0.57 ± 0.01 ^Bf^	0.55 ± 0.04 ^Bg^	0.65 ± 0.02 ^Bh^
Mercaptans (mg/100 g)	214.40 ± 0.79 ^Aa^	247.53 ± 41.47 ^Aa^	288.00 ± 1.58 ^Aab^	379.88 ± 75.03 ^Aab^	447.05 ± 81.11 ^Bab^	420.32 ± 38.69 ^Bc^	652.76 ± 39.10 ^Bd^	619.73 ± 81.65 ^Bd^
**Nut**	Day 0	Day 2	Day 4	Day 6	Day 8	Day 10	Day 12	Day 14
pH	6.38 ± 0.01 ^Aa^	6.48 ± 0.01 ^Ab^	6.60 ± 0.01 ^Bc^	6.55 ± 0.01 ^Ad^	6.55 ± 0.01 ^Cd^	6.54 ± 0.01 ^Bd^	6.62 ± 0.01 ^Cc^	6.72 ± 0.01 ^Ce^
Moisture (g/100 g)	73.76 ± 1.04 ^Aa^	72.36 ± 0.83 ^Aa^	72.49 ± 1.06 ^ABa^	71.86 ± 0.93 ^Ba^	71.37 ± 0.87 ^Ba^	70.90 ± 1.03 ^Bb^	70.49 ± 0.93 ^Bab^	70.06 ± 1.25 ^Bab^
L*	43.23 ± 0.47 ^Aa^	47.41 ± 0.79 ^Ca^	41.68 ± 2.56 ^Ba^	40.76 ± 0.17 ^Ba^	41.68 ± 2.56 ^Aa^	37.49 ± 1.06 ^Cb^	36.58 ± 0.30 ^Bab^	37.28 ± 7.88 ^Aab^
a*	6.76 ± 0.98 ^Aa^	4.14 ± 3.41 ^Aa^	6.77 ± 1.45 ^ABa^	6.04 ± 0.29 ^Aa^	8.10 ± 2.03 ^Aa^	7.34 ± 0.27 ^Ca^	13.92 ± 0.37 ^Cb^	3.81 ± 2.19 ^Aa^
b*	6.52 ± 0.32 ^Aa^	3.35 ± 0.94 ^Ba^	7.62 ± 1.73 ^Aab^	5.74 ± 0.17 ^Bab^	7.62 ± 1.73 ^Aab^	3.31 ± 0.87 ^Aa^	8.41 ± 0.23 ^Cb^	3.88 ± 2.86 ^Aa^
Heme iron (μg/g)	19.13 ± 0.01 ^Aa^	17.84 ± 0.01 ^Cb^	16.67 ± 0.01 ^Cc^	16.10 ± 0.01 ^Cd^	15.08 ± 0.01 ^Ce^	15.65 ± 0.01 ^Cf^	13.82 ± 0.01 ^Cg^	12.62 ± 0.01 ^Ch^
Hemoglobin (AU)	5.06 ± 0.01 ^Aa^	4.34 ± 0.01 ^Cb^	4.57 ± 0.01 ^Cb^	3.82 ± 0.01 ^Cc^	3.10 ± 0.01 ^Cd^	4.12 ± 0.01 ^Cbc^	4.08 ± 0.01 ^Bbc^	2.57 ± 0.01 ^Ce^
TBARS (mg MDA/kg)	0.38 ± 0.00 ^Aa^	0.44 ± 0.01 ^Cb^	0.54 ± 0.02 ^Cc^	0.51 ± 0.01 ^Cd^	0.60 ± 0.02 ^Ce^	0.69 ± 0.04 ^Cf^	0.71 ± 0.01 ^Cg^	0.77 ± 0.01 ^Ch^
Mercaptans (mg/100 g)	214.40 ± 0.79 ^Aa^	287.71 ± 1.00 ^Aa^	372.71 ± 84.00 ^Aab^	459.00 ± 85.00 ^Aab^	543.73 ± 0.27 ^Bb^	612.18 ± 3.28 ^ACb^	676.26 ± 37.03 ^Bb^	870.20 ± 75.18 ^Abc^
**LEO**	Day 0	Day 2	Day 4	Day 6	Day 8	Day 10	Day 12	Day 14
pH	6.38 ± 0.01 ^Aa^	6.48 ± 0.01 ^Ab^	6.53 ± 0.02 ^Ac^	6.56 ± 0.01 ^ABcd^	6.54 ± 0.01 ^Dc^	6.54 ± 0.01 ^BCc^	6.64 ± 0.01 ^Ce^	6.72 ± 0.01 ^Cf^
Moisture (g/100 g)	73.76 ± 1.04 ^Aa^	74.55 ± 1.19 ^ABa^	73.98 ± 0.86 ^ABa^	72.07 ± 0.71 ^Ba^	70.52 ± 0.97 ^Aab^	69.45 ± 1.27 ^ABab^	68.91 ± 1.10 ^ABb^	68.78 ± 0.74 ^ABb^
L*	43.23 ± 0.47 ^Aa^	45.21 ± 0.80 ^Da^	39.39 ± 0.59 ^Aab^	35.06 ± 0.37 ^Cab^	42.33 ± 3.94 ^Ba^	39.60 ± 0.35 ^Dac^	44.17 ± 0.16 ^Ca^	38.92 ± 1.45 ^Aab^
a*	6.76 ± 0.98 ^Aa^	5.51 ± 0.22 ^Aa^	6.11 ± 0.03 ^ABa^	5.46 ± 0.08 ^ABa^	6.95 ± 0.08 ^Aa^	8.65 ± 0.13 ^Bb^	7.44 ± 3.61 ^Dab^	8.28 ± 1.07 ^Bab^
b*	6.52 ± 0.32 ^Aa^	1.74 ± 0.89 ^BCb^	5.14 ± 0.08 ^Aa^	4.21 ± 0.13 ^Ca^	2.58 ± 3.38 ^ABb^	4.56 ± 0.11 ^Aa^	−0.71 ± 1.16 ^Db^	3.53 ± 0.39 ^Aa^
Heme iron (μg/g)	19.13 ± 0.01 ^Aa^	17.24 ± 0.01 ^Db^	14.81 ± 0.01 ^Dc^	12.59 ± 0.01 ^Dd^	14.54 ± 0.01 ^De^	11.94 ± 0.01 ^Df^	10.59 ± 0.01 ^Dg^	9.60 ± 0.01 ^Dh^
Hemoglobin (AU)	5.06 ± 0.01 ^Aa^	4.49 ± 0.01 ^Db^	4.53 ± 0.01 ^Dc^	3.43 ± 0.01 ^Dd^	3.78 ± 0.01 ^De^	3.37 ± 0.01 ^Df^	2.79 ± 0.01 ^Cg^	2.23 ± 0.01 ^Dh^
TBARS (mg MDA/kg)	0.38 ± 0.00 ^Aa^	0.46 ± 0.03 ^Db^	0.59 ± 0.02 ^Dc^	0.68 ± 0.05 ^Dd^	0.79 ± 0.10 ^De^	0.71 ± 0.03 ^Df^	0.85 ± 0.00 ^Dg^	1.06 ± 0.02 ^Dh^
Mercaptans (mg/100 g)	214.40 ± 0.79 ^Aa^	330.55 ± 42.70 ^Aa^	473.35 ± 102.32 ^ABab^	535.38 ± 84.94 ^Aab^	488.91 ± 36.69 ^BCab^	748.17 ± 50.83 ^Db^	901.91 ± 62.41 ^ACb^	1010.75 ± 57.14 ^Abc^

C-C: cinnamon and clove powder. Nut: nutmeg powder. LEO: lavender essential oil. MDA: malonic dialdehyde. Different uppercase letters in each column for each parameter indicate statistically significant (*p* < 0.05) differences among treatments according to Tukey’s honestly significant difference (HSD) test. Different lowercase letters in each row indicate statistically significant (*p* < 0.05) differences for each parameter during refrigerated storage according to Tukey’s HSD test.

**Table 4:** Correlation of hemoglobin absorbance values with heme iron content of buffalo meat during refrigerated storage.

Confidence Intervals				
Storage Time	Pearson Correlation	Sig. (Two-Tailed)	95% Confidence Intervals (Two-Tailed) ^a^	
			Lower	Upper
DAY0–DAY2	0.999	<0.001	0.996	0.999
DAY0–DAY4	0.993	<0.001	0.984	0.997
DAY0–DAY6	0.979	<0.001	0.951	0.991
DAY0–DAY8	0.992	<0.001	0.980	0.996
DAY0–DAY10	0.987	<0.001	0.970	0.994
DAY0–DAY12	0.983	<0.001	0.960	0.993
DAY0–DAY14	0.976	<0.001	0.945	0.990
DAY2–DAY4	0.998	<0.001	0.995	0.999
DAY2–DAY6	0.988	<0.001	0.972	0.995
DAY2–DAY8	0.996	<0.001	0.990	0.998
DAY2–DAY10	0.992	<0.001	0.981	0.997
DAY2–DAY12	0.991	<0.001	0.978	0.996
DAY2–DAY14	0.986	<0.001	0.967	0.994
DAY4–DAY6	0.996	<0.001	0.990	0.998
DAY4–DAY8	0.997	<0.001	0.992	0.999
DAY4–DAY10	0.996	<0.001	0.991	0.998
DAY4–DAY12	0.997	<0.001	0.993	0.999
DAY4–DAY14	0.995	<0.001	0.988	0.998
DAY6–DAY8	0.988	<0.001	0.971	0.995
DAY6–DAY10	0.993	<0.001	0.984	0.997
DAY6–DAY12	0.999	<0.001	0.997	0.999
DAY6–DAY14	0.998	<0.001	0.996	0.999
DAY8–DAY10	0.992	<0.001	0.981	0.996
DAY8–DAY12	0.990	<0.001	0.977	0.996
DAY8–DAY14	0.990	<0.001	0.977	0.996
DAY10–DAY12	0.997	<0.001	0.994	0.999
DAY10–DAY14	0.995	<0.001	0.989	0.998
DAY12–DAY14	0.999	<0.001	0.997	0.999

^a^ Estimation is based on Fisher’s r-to-z transformation.

**Table 5:** Correlation of TVC values with heme iron content of buffalo meat during refrigerated storage.

Confidence Intervals				
Storage Time	Pearson Correlation	Sig. (Two-Tailed)	95% Confidence Intervals (Two-Tailed) ^a^	
			Lower	Upper
DAY0–DAY2	0.997	<0.001	0.993	0.999
DAY0–DAY4	0.984	<0.001	0.963	0.993
DAY0–DAY6	0.942	<0.001	0.869	0.975
DAY0–DAY8	0.964	<0.001	0.918	0.985
DAY0–DAY10	0.938	<0.001	0.859	0.973
DAY0–DAY12	0.902	<0.001	0.784	0.957
DAY0–DAY14	0.814	<0.001	0.612	0.917
DAY2–DAY4	0.995	<0.001	0.987	0.998
DAY2–DAY6	0.964	<0.001	0.917	0.985
DAY2–DAY8	0.979	<0.001	0.950	0.991
DAY2–DAY10	0.956	<0.001	0.899	0.981
DAY2–DAY12	0.929	<0.001	0.840	0.969
DAY2–DAY14	0.850	<0.001	0.680	0.933
DAY4–DAY6	0.985	<0.001	0.966	0.994
DAY4–DAY8	0.989	<0.001	0.973	0.995
DAY4–DAY10	0.977	<0.001	0.947	0.990
DAY4–DAY12	0.961	<0.001	0.910	0.983
DAY4–DAY14	0.895	<0.001	0.769	0.954
DAY6–DAY8	0.974	<0.001	0.939	0.989
DAY6–DAY10	0.984	<0.001	0.963	0.993
DAY6–DAY12	0.989	<0.001	0.974	0.995
DAY6–DAY14	0.942	<0.001	0.869	0.975
DAY8–DAY10	0.971	<0.001	0.933	0.988
DAY8–DAY12	0.951	<0.001	0.888	0.979
DAY8–DAY14	0.898	<0.001	0.776	0.955
DAY10–DAY12	0.984	<0.001	0.963	0.993
DAY10–DAY14	0.933	<0.001	0.848	0.971
DAY12–DAY14	0.970	<0.001	0.931	0.987

^a^ Estimation is based on Fisher’s r-to-z transformation.

**Table 6:** Correlation of LAB values with mercaptan content of buffalo meat during refrigerated storage.

Confidence Intervals				
Storage Time	Pearson Correlation	Sig. (Two-Tailed)	95% Confidence Intervals (Two-Tailed) ^a^	
			Lower	Upper
DAY0–DAY2	0.977	<0.001	0.948	0.990
DAY0–DAY4	0.951	<0.001	0.888	0.979
DAY0–DAY6	0.969	<0.001	0.928	0.987
DAY0–DAY8	0.972	<0.001	0.936	0.988
DAY0–DAY10	0.961	<0.001	0.910	0.983
DAY0–DAY12	0.979	<0.001	0.951	0.991
DAY0–DAY14	0.968	<0.001	0.927	0.986
DAY2–DAY4	0.982	<0.001	0.959	0.992
DAY2–DAY6	0.993	<0.001	0.984	0.997
DAY2–DAY8	0.978	<0.001	0.948	0.990
DAY2–DAY10	0.975	<0.001	0.942	0.989
DAY2–DAY12	0.984	<0.001	0.963	0.993
DAY2–DAY14	0.979	<0.001	0.951	0.991
DAY4–DAY6	0.967	<0.001	0.925	0.986
DAY4–DAY8	0.946	<0.001	0.877	0.977
DAY4–DAY10	0.967	<0.001	0.925	0.986
DAY4–DAY12	0.975	<0.001	0.941	0.989
DAY4–DAY14	0.985	<0.001	0.965	0.994
DAY6–DAY8	0.968	<0.001	0.925	0.986
DAY6–DAY10	0.972	<0.001	0.936	0.988
DAY6–DAY12	0.982	<0.001	0.957	0.992
DAY6–DAY14	0.970	<0.001	0.931	0.987
DAY8–DAY10	0.959	<0.001	0.906	0.982
DAY8–DAY12	0.967	<0.001	0.923	0.986
DAY8–DAY14	0.962	<0.001	0.913	0.984
DAY10–DAY12	0.982	<0.001	0.959	0.992
DAY10–DAY14	0.990	<0.001	0.978	0.996
DAY12–DAY14	0.988	<0.001	0.973	0.995

^a^ Estimation is based on Fisher’s r-to-z transformation.

## Data Availability

The original contributions presented in this study are included in the article. Further inquiries can be directed to the corresponding author.
